# Virome metatranscriptomic profiling of birch pollen reveals a diverse viral community

**DOI:** 10.3389/fpls.2026.1897449

**Published:** 2026-08-31

**Authors:** Sahar Nouri, Denis Kutnjak, Anja Pecman, Hector Fernandez Colino, Carmen Büttner, Susanne von Bargen, Artemis Rumbou

**Affiliations:** 1 Division of Phytomedicine, Albrecht Daniel Thaer Institute of Agricultural and Horticultural Sciences, Faculty of Life Sciences, Humboldt-Universität zu Berlin, Berlin, Germany; 2 Department of Biotechnology and Systems Biology, National Institute of Biology, Ljubljana, Slovenia

**Keywords:** *Betula*, idaeovirus, nepovirus, totivirus, pollen virome, urban ecosystems

## Abstract

**Introduction:**

Viruses are increasingly recognized as integral components of plant-associated biological systems. However, their occurrence and diversity in the reproductive tissues of woody plants remain poorly understood. Birch (*Betula* spp.) produces large quantities of wind-dispersed pollen that can travel over long distances and may harbour viruses or virus-derived nucleic acids originating from the host plant and/or its associated microbiota.

**Methods:**

We investigated the virome of birch pollen collected from trees growing in central and suburban Berlin, Germany. Metatranscriptomic analyses were performed on pooled pollen samples collected in 2020. These analyses were complemented by RT-PCR screening of individually processed pollen samples collected in 2025 from resampled trees. Primer walking was additionally used to recover an extended genome sequence of a pollen-associated birch toti-like virus.

**Results:**

Multiple virus-associated contigs were identified in both pooled metatranscriptomic datasets. These included sequences corresponding to the cherry leaf roll virus (CLRV), the birch idaeovirus (BIV) and the birch toti-like virus (BTLV), as well as additional virus-like contigs provisionally assigned to lineages related to the *Orthototiviridae, Botourmiaviridae, Endornaviridae*, and *Chrysoviridae* families. RT-PCR analysis of individually processed pollen samples confirmed the continued detection of CLRV, BIV, and BTLV within the Berlin sampling framework. A near-complete genome sequence was recovered from a pollen-derived BTLV isolate from Berlin, showing high amino acid sequence identity to a recently described leaf-derived BTLV isolate from the United States.

**Discussion:**

These findings demonstrate that birch pollen harbours a diverse assemblage of plant- and/or microbiome-associated viruses and expand the known tissue distribution and geographic range of BTLV. More broadly, they establish pollen as an underexplored ecological niche for virome research and provide a foundation for future studies on the ecology, transmission dynamics, and epidemiological significance of pollen-associated and pollen-transmitted viruses.

## 1 Introduction

Viruses are widespread in plants and plant-associated environments, playing important roles in plant health, disease development, and ecosystem dynamics (Roossinck, 2015; Jones, 2021). Many viruses infect woody perennial hosts in both natural and managed systems, including forests and urban green spaces, where infections may remain latent, persist chronically, or be associated with severe decline depending on the host, the virus, and the environmental context (Büttner et al., 2023).

High-throughput sequencing (HTS) has substantially expanded our understanding of virus diversity in trees, including birch, revealing that the viromes of woody hosts are often considerably more complex than suggested by symptom-based diagnostic approaches (Rumbou et al., 2021; Vainio et al., 2024). To date, most tree virome studies have focused on vegetative tissues, particularly leaves and bark, whereas reproductive structures have received comparatively little attention (Bradamante et al., 2021).

This knowledge gap is particularly relevant in birch (*Betula* spp.), an anemophilous genus that produces large amounts of lightweight pollen capable of long-distance atmospheric dispersal, potentially even across national borders (Ranta et al., 2008; Sofiev et al., 2013). Pollen is not only the male reproductive structure of flowering plants but also a biologically complex microhabitat that harbors diverse microbial communities, including bacteria, fungi and other microorganisms (Hodgson et al., 2014; Shevtsova et al., 2016; Obersteiner et al., 2016; Ezike et al., 2016). As these pollen-associated microorganisms may themselves host viruses, particularly mycoviruses, pollen has the potential to represent a composite reservoir of both plant- and microbiome-associated viral diversity (Hough et al., 2023; Ezawa et al., 2023). Despite this ecological significance, the viromes of pollen remain poorly characterized, limiting our understanding of virus ecology, and potential transmission within reproductive tissues and the surrounding environment (Lee and Jeong, 2022; Fetters and Ashman, 2023; Fetters et al., 2025; Pagán, 2022).

Metatranscriptomic surveys have shown that the known birch virome comprises viruses belonging to at least 8 genera: *Badnavirus, Nepovirus, Carlavirus, Idaeovirus, Totivirus, Capillovirus, Ilarvirus, Necrovirus* (Rumbou et al., 2021). Birch viromes are typically characterized by mixed infections involving multiple viral taxa and variants of the same virus species (Rumbou et al., 2020). Among the viruses most frequently reported from urban birch populations are birch leaf roll-associated virus (BLRaV; *Badnavirus volubetulae*), cherry leaf roll virus (CLRV; *Nepovirus avii*), apple mosaic virus (ApMV, *Ilarvirus ApMV*), birch carlavirus (BCV, *Carlavirus betulae*), and birch idaeovirus (BIV) (Büttner et al., 2023). Of these viruses, CLRV is well-established as both seed- and pollen-borne in birch (Rebenstorf et al., 2006; Rumbou et al., 2009). Regarding BIV, although it has been previously detected in birch pollen, its natural transmission route remains unresolved (Gilles et al., 2023).

Previous studies of birch pollen viromes have largely relied on targeted molecular assays, demonstrating that the reproductive tissue of birch can harbor known viral agents (Gilles et al., 2023). However, these targeted approaches were restricted to a limited number of expected viruses and therefore did not capture the broader diversity of virus-associated sequences potentially present in birch pollen. To comprehensively characterize the virome associated with *Betula* sp. pollen in the metropolitan area of Berlin, Germany, we applied an untargeted metatranscriptomic approach. This enabled the unbiased detection and characterization of both known and previously unrecognized viruses and virus-like sequences associated with the birch pollen microhabitat.

## 2 Materials and methods

### 2.1 Sample collection

Birch pollen samples were collected from *Betula* sp. trees in Berlin, Germany, during their flowering seasons in 2020 and 2025. For HTS, two pooled pollen samples collected in 2020 were analyzed: one representing trees from central Berlin (districts of Friedrichshain, Köpenick, Kreuzberg, Mitte, Neukölln, Pankow, Tempelhof, Treptow), comprising pollen from 14 trees, and a second representing the Berlin-Zehlendorf district, comprising pollen from 11 trees. At the time of sampling, all trees were visually inspected for virus-like foliar symptoms. Trees exhibiting chlorotic spots, mosaic, interveinal chlorosis, leaf rolling or leaf deformation were classified as symptomatic, whereas those without visible symptoms were classified as apparently asymptomatic.

For RT-PCR-based *post-hoc* confirmation, pollen was collected in 2025 from individually processed trees within the same Berlin sampling framework as the pooled samples analyzed by HTS in 2020. Of the 14 trees originally included in the Berlin-Center pool, 11 were successfully resampled, while eight of the 11 trees from the Berlin-Zehlendorf pool could be resampled. In total, pollen samples were collected from 19 individual trees (**Supplementary Table 1**). The remaining six trees could not be included because suitable flowering male catkins were unavailable and the tree crowns were not safely accessible; in one case in Berlin-Zehlendorf, one tree had been removed. For each tree, pollen was collected from multiple male catkins, with at least 10 catkins sampled per tree to minimize within-tree variability. Catkins were collected during flowering at growth stages 56–59 of the extended BBCH phenological scale, before large-scale pollen release, to minimize incidental deposition of ambient pollen. All samples were transported to the laboratory immediately after collection and processed without delay.

### 2.2 Catkin drying and pollen isolation

Prior to pollen extraction, male catkins were stored in sealed, heat-resistant containers using silica gel orange desiccant (Carl Roth GmbH + Co. KG, Karlsruhe, Germany) until fully desiccated. Pollen was released by vigorous shaking and tapping of the dried catkins under clean laboratory conditions and subsequently enriched by sequential dry sieving through sterilized 100 µm and 70 µm meshes. The resulting pollen fraction was aliquoted into RNase-free microcentrifuge tubes to avoid repeated freeze-thaw cycles and placed at -20 °C for short-term or -80 °C for long-term preservation until RNA extraction.

### 2.3 RNA extraction, library preparation and Illumina sequencing

Total RNA was extracted from pollen using a silica-based method. Briefly, approximately 300 mg pollen was transferred to bead-beating tubes containing 700 µL grinding buffer, composed of 6 M guanidine hydrochloride, 0.2 M sodium acetate pH 5.2, 25 mM EDTA, 1 M potassium acetate, and 2.5% (w/v) polyvinylpyrrolidone (PVP-40), followed by the addition of 200 µL 10% (w/v) sodium dodecyl sulfate to facilitate lysis. Samples were incubated in a thermomixer at 70°C for 10 min with shaking at 800 rpm, followed by centrifugation at 15,000 × g for 10 min at 4 °C. The clarified supernatant was transferred to a fresh RNase-free tube and processed according to Boom et al. (1990). RNA was eluted in RNase-free water, and concentration was measured using a NanoDrop One spectrophotometer (Thermo Fisher Scientific, Waltham, MA, USA).

After DNase digestion, ribosomal RNA was depleted from total RNA extracted from the pooled pollen samples using the RiboMinus™ Plant Kit for RNA-Seq (Invitrogen, Thermo Fisher Scientific, Waltham, MA, USA). The rRNA-depleted RNA was used for double-stranded cDNA synthesis with random hexamer primers. First-strand cDNA synthesis was performed using 4× First Strand Reaction Mix and First Strand Enzyme Mix, followed by second-strand synthesis using 5× Second Strand Reaction Mix and Second Strand Enzyme Mix (Invitrogen, Thermo Fisher Scientific, Waltham, MA, USA), according to the manufacturer’s instructions. Library preparation and sequencing of the pooled pollen datasets Berlin-Center and Berlin-Zehlendorf were performed by BaseClear B.V. (Leiden, The Netherlands). Sequencing libraries were prepared from double-stranded cDNA using the Nextera XT DNA Library Preparation Kit (Illumina, San Diego, CA, USA) and were sequenced on an Illumina NovaSeq 6000 platform using paired-end 150-bp reads (2 × 150 bp).

### 2.4 Transcriptome assembly and virus identification

Raw paired-end reads were quality assessed and processed using CLC Genomics Workbench v25.0.1 (QIAGEN, Hilden, Germany). Adapter sequences were removed, reads were quality-trimmed using a quality limit of 0.05 (approximately corresponding to a PHRED score of 14), and reads shorter than 30 bp were discarded. The resulting high-quality reads were initially screened for viral sequences by mapping against the NCBI Viral RefSeq-based reference database (release of January 8, 2025).

Trimmed reads were independently assembled using both the *de novo* assembler implemented in CLC Genomics Workbench and SPAdes v4.0.0 in rnaviralSPAdes mode with default parameters. The CLC workflow was used for the initial identification of virus-associated sequences through Viral RefSeq mapping and complementary Pfam-based conserved-domain searches to detect divergent virus-like sequences. For selected viral reference matches, the total number of mapped reads, number of properly paired reads, consensus sequence length, reference genome length, and mean sequencing depth were extracted from the CLC mapping output. These CLC-derived reference-based mapping statistics were evaluated independently of the SPAdes-derived contig coverage values and are presented in **Supplementary Table 4**.

SPAdes-derived contigs were used for all subsequent downstream analyses, including taxonomic classification (in MEGAN), sequence characterization (in BLASTn and BLASTx), contig coverage assessment (in SPAdes), and sequence submission to GenBank.

Translated similarity searches of the SPAdes-derived contigs were performed using DIAMOND v0.9.14 (Buchfink et al., 2015), in sensitive mode against the NCBI non-redundant (nr) protein database (release on November 21, 2024). The resulting alignments were imported into MEGAN v6.25.10 (Huson et al., 2016), where taxonomic assignments were performed using the lowest common ancestor (LCA) algorithm. Candidate contigs were retained if they received a virus-associated taxonomic assignment by DIAMOND/MEGAN and/or exhibited significant identity to viral nucleotide or protein sequences in BLASTn or BLASTx searches, including conserved viral protein domains. For each virus or virus-like sequence, the longest representative contig was selected for downstream analyses. Where multiple contigs represented different genomic segments or coding regions, representative contigs from each segment were retained to capture the genomic diversity of the detected virus-associated sequences.

Contigs represented by short sequence fragments and supported by low SPAdes assembly coverage were interpreted conservatively and classified as provisional virus-like signals rather than confirmed viral sequences. To assess sequence support, the quality-trimmed reads were mapped back to the representative viral and virus-like contigs.

Comparisons between the two pooled HTS datasets were descriptive only. Because each sampling location was represented by a single pooled sequencing library, the pools differed in the number of contributing trees, and sequencing depth varied between datasets, no formal statistical analyses were performed. Consequently, differences in the number, abundance, or diversity of virus-associated contigs detected between the Berlin-Center and Berlin-Zehlendorf datasets were only interpreted qualitatively.

### 2.5 Post-hoc RT-PCR confirmation and amplification of near-complete BTLV isolate

Virus-specific RT-PCR assays were performed on individually processed birch pollen samples collected in 2025 from resampled trees, where possible, within the same Berlin sampling framework as the pooled samples analyzed by HTS in 2020. Because archived RNA from individual trees sampled in 2020 was not available, the 2025 RT-PCR assays served as a *post-hoc* confirmation of selected virus targets in birch pollen rather than as a direct technical validation of the original HTS detections.

Complementary DNA (cDNA) was synthesized from total RNA using random hexamer primers and RevertAid Reverse Transcriptase (Thermo Fisher Scientific, Waltham, MA, USA). PCR amplification was performed using DreamTaq DNA Polymerase according to the manufacturer’s instructions (Thermo Fisher Scientific, Waltham, MA, USA). Differences in RT-PCR detection frequencies between Berlin-Center and Berlin-Zehlendorf sampling sites were evaluated using two-sided Fisher’s exact tests. Exact two-sided *P* values are reported, and results were interpreted cautiously because of the small sample size.

To compare RT-PCR detection in pollen with previously obtained RT-PCR results from leaf samples of the corresponding trees, leaf RT-PCR data were retrieved from previous studies and unpublished datasets (Opoku, 2023; personal communication). The occurrence of BLRaV, BiCV, and ApMV in leaf samples, together with the corresponding foliar symptoms observed at the time of pollen sampling, is summarized in **Supplementary Table 1**.

To further characterize birch toti-like virus (BTLV), the isolate E59142, obtained from *Betula* sp. pollen that was collected in Berlin-Zehlendorf in April 2025, was selected for primer walking. The aim was to resolve ambiguous regions in the HTS-derived BTLV assembly and to obtain a near-complete genome sequence representative of this location. Virus-specific primers were designed from a multiple sequence alignment comprising BTLV-associated contigs derived from the pooled HTS dataset together with the available BTLV reference genome (GenBank accession no. PP740463). These primers were used to amplify overlapping genomic fragments (**Supplementary Table 2**), which were cloned into the pGEM-T Easy vector (Promega, Madison, WI, USA) and transformed into chemically competent *Escherichia coli* XL1-Blue MRF´ cells (Agilent Technologies, Santa Clara, CA, USA). Recombinant colonies were screened by colony PCR using the vector-specific M13F/M13R vector primers (Promega). For sequence verification, at least two recombinant colonies per amplicon were selected for plasmid extraction using the NucleoSpin Plasmid EasyPure kit (Macherey-Nagel, Düren, Germany). Purified plasmids were sequenced bidirectionally using vector-specific primers by Sanger sequencing (Macrogen Europe, Amsterdam, The Netherlands).

### 2.6 Phylogenetic analyses of virus-associated contigs

Phylogenetic analyses were restricted to CLRV-associated contigs, as CLRV was represented by the greatest number of contigs in both pooled datasets and showed substantial sequence diversity. In contrast, BIV and BTLV were represented by relatively few overlapping contigs and the limited availability of closely related reference sequences precluded robust phylogenetic analyses. Likewise, the provisional virus-like contigs were short and supported by low sequencing coverage and were therefore considered unsuitable for reliable phylogenetic inference.

Phylogenetic analysis was undertaken to evaluate the relationships among CLRV-associated contigs and to assess whether overlapping contigs represented assembly fragments of the same viral segment or distinct CLRV segments. Because individual contigs may originate from different regions of the same viral genome segment, the number of contigs was not interpreted as the number of viral variants. Instead, overlapping contigs derived from the same genomic RNA were compared based on their genomic position, pairwise nucleotide identity, and phylogenetic placement. Contigs sharing overlapping genomic coordinates, high nucleotide identity, and clustering within the same phylogenetic lineage were interpreted as representing the same or closely related CLRV variants. Conversely, overlapping contigs with lower nucleotide identity and/or placement in different phylogenetic clades or well-supported subclades were considered evidence for distinct CLRV variants within a pooled sample. Because the HTS libraries were generated from pooled pollen samples, and RNA1 and RNA2 contigs could not be assigned to individual trees or linked into complete viral haplotypes, the exact number of CLRV variants or genotypes present in each pool could not be determined.

Reference sequences used for representative CLRV isolates and tomato ringspot virus (ToRSV) outgroup, were retrieved from GenBank. Multiple sequence alignments were generated using MAFFT v7 (Katoh and Standley, 2013) with default parameters. Maximum-likelihood phylogenetic trees were constructed in MEGA 12 (Kumar et al., 2024) using 1,000 bootstrap replicates and included representative CLRV isolates from diverse hosts and geographic origins. The optimal nucleotide substitution model for each dataset was selected according to the lowest Bayesian Information Criterion (BIC) score. The General Time Reversible model with gamma-distributed rate variation and a proportion of invariant sites (GTR+G+I) was selected for RNA1, whereas the Kimura two-parameter model with gamma-distributed rate variation and a proportion of invariant sites (K2+G+I) was selected for RNA2. The RNA1 and RNA2 trees were rooted with the corresponding ToRSV RNA1 and RNA2 sequences, respectively, as outgroups because ToRSV is a closely related nepovirus outside the CLRV ingroup. Trees are presented as bootstrap consensus trees, and only bootstrap support values ≥50% are shown.

## 3 Results

### 3.1 HTS output and *de novo* assembly statistics

High-throughput sequencing of pooled birch pollen samples collected from two urban locations in Berlin generated two pooled metatranscriptomic datasets representing Berlin-Center and Berlin-Zehlendorf. Following quality filtering, 17,147,307 paired-end reads were retained for the Berlin-Center dataset and 13,658,939 paired-end reads for the Berlin-Zehlendorf dataset. A summary of sequencing output for both pooled pollen datasets is provided in **Supplementary Table 3**.

### 3.2 Detection of virus and virus-like sequences in pooled birch pollen datasets

Virus and virus-like sequences reported in this study were derived from the SPAdes *de novo* assemblies. The characteristics of the selected virus-associated contigs, together with their corresponding GenBank accession numbers, are summarized in **Tables 1**, **2**. *De novo* assembly using SPAdes resulted in 39,803 contigs and 28,081 contigs for the Berlin-Center and Berlin-Zehlendorf datasets, respectively. High-confidence virus-associated contigs are summarized in **Tables 3**, **4** and formed the basis for all subsequent analyses, including contig characterization, BLAST analyses, coverage assessment, and sequence submission.

**Table 1:** Representative virus-associated contigs identified in pooled birch pollen from Berlin-Center and their BLASTn and BLASTx similarities to reference viral sequences.

Virus-associated contig	GenBank accession no.	Encoded genomic region/protein	Sequence length (nt)*	BLASTn identity (%)	BLASTn E-value	Top BLASTn hit (GenBank accession no.)	BLASTx identity (%)	Aligned length (aa)	Top BLASTx hit (protein accession no.)	Assigned family	BLASTx E-value
birch-associated alphachrysovirus-like 21403	PZ525086	dsRNA4/P4	289	No significant similarity found	–	–	36	95	Macrophomina phaseolina chrysovirus 1 (YP_009667011.1)	*Chrysoviridae*	1e-19
birch-associated toti-like virus 11152	PZ435551	RdRP	452	76.50	8e-16	*Ghabrivirales* sp. (PV236722)	67.33	150	Hortaea werneckii totivirus 1 (AZT88647.1)	*Orthototiviridae*	1e-66
birch-associated toti-like virus 4326	PZ435552	RdRP	993	73.31	9e-18	*Ghabrivirales* sp. (PV236722)	65	304	Hortaea werneckii totivirus 1 (AZT88647.1)	*Orthototiviridae*	0.0
birch toti-like virus 33227	PZ525087	RdRP	239	91.85	7e-88	birch toti-like virus (PP740463.1)	91.14	79	birch toti-like virus (XAI71506.1)	*Orthototiviridae*	5e-42
birch toti-like virus 5268	PZ525088	CP	853	95	0.0	birch toti-like virus (PP740463.1)	98.89	198	birch toti-like virus (XAI71505.1)	*Orthototiviridae*	0.0
birch-associated alphaendornavirus-like 34971	PZ525089	RdRP	233	79.56	1e-16	Sichuan alphaendornavirus 2 (MW897169.1)	79.03	62	Sichuan alphaendornavirus 2 (QYF50052.1)	*Endornaviridae*	3e-25
birch idaeovirus 48	PZ435520	RdRP	5,433	94.25	0.0	birch idaeovirus (PQ867863.1)	100	1,691	birch idaeovirus (QDX18419.1)	*Mayoviridae*	0.0
birch idaeovirus 820	PZ435521	MP	2,321	93	0.0	birch idaeovirus (PQ867864.1)	100	362	birch idaeovirus (QDX18420.1)	*Mayoviridae*	0.0
birch-associated ourmia-like virus 27774	PZ435554	RdRP	258	95.74	3e-116	Erysiphe necator associated ourmia-like virus 26 (MN611554.1)	100	85	Erysiphe necator associated ourmia-like virus 26 (QKI79856.1)	*Botourmiaviridae*	6e-50
birch-associated ourmia-like virus 23512	PZ525090	RdRP	277	92	6e-107	Plasmopara viticola lesion associated ourmia-like virus 70 (NC_076799.1)	98.90	91	Plasmopara viticola lesion associated ourmia-like virus 70 (YP_010800264.1)	*Botourmiaviridae*	2e-54
cherry leaf roll virus 218	PZ435527	RNA1/polyprotein	3,508	92	0.0	cherry leaf roll virus (LT883167.1)	99	1,168	cherry leaf roll virus (SMZ59133.1)	*Secoviridae*	0.0
cherry leaf roll virus 237	PZ435529	RNA2/polyprotein	3,429	90.18	0.0	cherry leaf roll virus (LT883166.1)	93.56	1,113	cherry leaf roll virus (SMZ59134.1)	*Secoviridae*	0.0

CP: coat protein; MP: movement protein; RdRP: RNA-dependent RNA polymerase.

**Table 2:** Representative virus-associated contigs identified in pooled birch pollen from Berlin-Zehlendorf and their BLASTn and BLASTx similarities to reference viral sequences.

Virus-associated contig	GenBank accession no	Encoded genomic region/protein	Sequence length (nt)*	BLASTn identity (%)	BLASTn E-value	Top BLASTn hit (GenBank accession no.)	BLASTx identity (%)	Aligned length (aa)	Top BLASTx hit (protein accession no.)	Assigned family	BLASTx E-value
birch toti-like virus 12804	PZ525091	CP	314	94.27	4e-131	birch toti-like virus (PP740463.1)	97.12	104	birch toti-like virus (XAI71505.1)	*Orthototiviridae*	1e-61
birch idaeovirus 25	PZ435518	RdRP	4,402	99.46	0.0	birch idaeovirus (MK402235.1)	99.93	1,348	birch idaeovirus (QDX18419.1)	*Mayoviridae*	0.0
birch idaeovirus 365	PZ435519	MP	2,326	*99*	0.0	birch idaeovirus isolate C502 (PQ867864.1)	100	362	birch idaeovirus (QDX18420.1)	*Mayoviridae*	0.0
birch ourmia-like virus 14815	PZ435553	RdRP	233	95.11	9e-94	Erysiphe necator associated ourmia-like virus 26 (MN611554.1)	100	95	Erysiphe necator associated ourmia-like virus 40 (QKI79870)	*Botourmiaviridae*	7e-57
cherry leaf roll virus 11	PZ435522	Polyprotein 1	5,110	92.61	0.0	cherry leaf roll virus (MK402281.1)	98.65	1,701	cherry leaf roll virus (QYA72483.1)	*Secoviridae*	0.0
cherry leaf roll virus 42	PZ435526	polyprotein 2	3,926	90.78	0.0	cherry leaf roll virus (MK402282.1)	94.85	1,262	cherry leaf roll virus (AFB82732.1)	*Secoviridae*	0.0

CP: coat protein; MP: movement protein; RdRP: RNA-dependent RNA polymerase.

**Table 3:** Virus and virus-like contigs identified from pooled birch pollen metatranscriptomes from Berlin-Center and their assembly characteristics.

No.	Virus or virus-like sequence	Assigned family	No. of SPAdes derived contigs	SPAdes contig coverage range	Minimum contig length (nt)	Maximum contig length (nt)
1	birch-associated alphachrysovirus-like virus	*Chrysoviridae*	1	1,44	289	289
2	birch-associated toti-like virus 4326	*Orthototiviridae*	1	2,72	993	993
3	birch-associated toti-like virus 11152	*Orthototiviridae*	1	2,05	452	452
4	birch toti-like virus (BTLV)	*Orthototiviridae*	5	1,28-580,79	239	853
5	birch alphaendornavirus-like virus	*Endornaviridae*	1	2,93	233	233
6	birch idaeovirus (BIV)	*Mayoviridae*	2	206,87-382,68	2,321	5,433
7	birch-associated ourmia-like virus	*Botourmiaviridae*	2	0,7-2,52	258	277
8	cherry leaf roll virus (CLRV)	*Secoviridae*	36	1,13-291,82	142	3,508

**Table 4:** Virus and virus-like contigs identified from pooled birch pollen metatranscriptomes from Berlin-Zehlendorf and their assembly characteristics.

No.	Virus or virus-like sequence	Assigned family	No. of contigs	SPAdes contig coverage range	Minimum contig length (nt)	Maximum contig length (nt)
1	birch toti-like virus (BTLV)	*Orthototiviridae*	1	3,1	314	314
2	birch idaeovirus (BIV)	*Mayoviridae*	7	1,01-457,87	138	4,402
3	birch-associated ourmia-like virus	*Botourmiaviridae*	1	0,97	233	233
4	cherry leaf roll virus (CLRV)	*Secoviridae*	8	19,70-65,36	145	5,110

HTS analysis of pooled birch pollen samples revealed assemblage of virus and virus-like sequences in both datasets. The Berlin-Center dataset contained contigs assigned to eight virus or virus-like sequence groups: CLRV, BIV, BTLV, two birch-associated toti-like viruses, a birch ourmia-like virus, a birch alphachrysovirus-like virus, and a birch alphaendornavirus-like virus (**Table 3**). In contrast, the Berlin-Zehlendorf dataset contained contigs corresponding to CLRV, BIV, BTLV, and a birch-associated ourmia-like sequence, representing a lower diversity of virus-associated sequence groups. This difference may partly reflect the lower sequencing depth of the Berlin-Zehlendorf library, which yielded less quality-filtered paired-end reads compared with the reads of the Berlin-Center dataset.

Reference-based CLC Viral RefSeq mapping (**Supplementary Table 4**) supported the presence of the principal virus-associated signals detected by the SPAdes assemblies. CLRV showed the strongest read support in both pooled pollen datasets, with substantially higher numbers of mapped reads, longer consensus sequences, greater reference coverage, and higher mean read depth in the Berlin-Center dataset than in the Berlin-Zehlendorf dataset. The BIV-associated signal was represented by partial RNA1 consensus sequences of 568 nt (Berlin-Center) and 645 nt (Berlin-Zehlendorf), supported by relatively few mapped reads. The BTLV-associated signal yielded only short partial consensus sequences (298–669 nt) when mapped against related Panax notoginseng virus A and B reference genomes, with considerably greater read support in the Berlin-Center dataset than in the Berlin-Zehlendorf dataset (**Supplementary Table 4**).

BLASTn and BLASTx analyses confirmed that the CLRV contigs detected by HTS were related to both genomic RNA segments of CLRV. Contigs corresponding to CLRV RNA1 and RNA2 were recovered from both pooled birch pollen datasets, indicating the occurrence of CLRV-associated sequences in samples from both Berlin-Center and Berlin-Zehlendorf. In the Berlin-Center dataset, the RNA1-associated contig CLRV-218 (GenBank acc. no. PZ435527; 3,508 nt) shared 92% nt (nucleotide) identity and 99% aa (amino acid) identity with a CLRV isolate from Berlin, Germany (GenBank acc. no. LT883167.1). The RNA2-associated contig CLRV-237 (PZ435529; 3,429 nt) shared 90.18% nt and 93.56% aa identity with the corresponding CLRV Berlin isolate (**Table 1**). In the Berlin-Zehlendorf dataset, the RNA1-associated contig CLRV-11 (PZ435522; 5,110 nt) shared 92.61% nt and 98.65% aa identity to the same CLRV Berlin isolate. The RNA2-associated contig CLRV-42 (PZ435526; 3,926 nt) shared 90.78% nt and 94.85% aa identity with a CLRV isolate from the United States (**Table 2**).

Pairwise sequence comparisons revealed substantial diversity among the CLRV-associated contigs. The RNA1 contigs CLRV-11 from Berlin-Zehlendorf (GenBank acc. no. PZ435522) and CLRV-218 Berlin-Center (PZ435527) shared 90.91% nt identity and 99.64% aa identity (data not shown). Within the Berlin-Center dataset, the lowest RNA1 sequence identity was observed between CLRV-1474 (PZ435535; 1,808 nt) and CLRV-1746 (PZ435538; 1,628 nt), which shared 78% nt and 89.12% aa identity. For RNA2, CLRV-42 from Berlin-Zehlendorf (PZ435526) and CLRV-322 from Berlin-Center (PZ435530; 3,186 nt) shared 88% nt and 94% aa identity. The lowest pairwise identity within the Berlin-Center was detected between CLRV-4276 (PZ435542; 999 nt) and CLRV-4661 (PZ435543; 935 nt), with 76% nt and 82.40% aa identity.

These comparisons demonstrate that the recovered CLRV-associated contigs represent multiple genetically distinct CLRV genotypes, than a single homogeneous virus isolate. In the Berlin-Center dataset, the low nucleotide identities among overlapping RNA1 and RNA2 contigs, together with their phylogenetic placement (see below), support the presence of at least two divergent RNA1 and RNA2 sequence types. However, because the HTS library was generated from pooled pollen and the RNA1 and RNA2 contigs could not be assigned to individual trees or linked into complete viral genomes, the exact number of CLRV variants could not be determined. The Berlin-Zehlendorf dataset confirmed the presence of both CLRV RNA segments RNA1 and RNA2, which suggests that only one CLRV variant is present in the dataset.

BIV-associated contigs were detected in both pooled birch pollen datasets. Two BIV contigs were identified in the Berlin-Center dataset (**Table 3**), whereas seven BIV contigs were detected in the Berlin-Zehlendorf dataset (**Table 4**). In Berlin-Center, the RNA1/RdRP-associated contig BIV-48 (GenBank acc. no. PZ435520; 5,433 nt) showed 94.25% nt identity with a birch idaeovirus isolate (PQ867863.1) and 100% aa identity with the corresponding the RNA-dependent RNA polymerase (RdRP) from the BIV isolate obtained from *Betula pendula* in Berlin, Germany (QDX18419.1) (**Table 1**). The RNA2/movement protein-associated contig BIV-820 (PZ435521; 2,321 nt) showed 93% nt identity with the only available BIV-sequence from birch idaeovirus (PQ867864.1) and 100% aa identity with the BIV movement protein of the same Berlin isolate (QDX18420.1) (**Table 1**).

In the Berlin-Zehlendorf dataset, the RNA1/RdRP-associated contig BIV-25 (acc. no. PZ435518; 4,402 nt) showed 99.46% nt identity (MK402235.1) and 99.93% aa identity to the BIV RdRP (QDX18419.1) (**Table 2**). The RNA2/movement-protein-associated contig BIV-365 (PZ435519; 2,326 nt) showed 99% nt identity to the BIV isolate C502 (PQ867864.1) and 100% aa identity to the BIV movement protein from the same *B. pendula* in Berlin, Germany (QDX18420.1) (**Table 2**).

Pairwise alignment comparisons of BIV contigs between the two datasets revealed very high sequence identity. The RNA1-associated contigs BIV-25 from Berlin-Zehlendorf (acc. no. PZ435518) and BIV-48 from Berlin-Center (acc. no. PZ435520) shared 99% nt identity and 100% aa identity (data not shown). Similarly, the RNA2-associated contigs BIV-365 from Berlin-Zehlendorf RNA2 (acc. no. PZ435519) and BIV-820 from Berlin-Center (acc. no. PZ435521) shared 99% nt identity and 100% aa identity. These results indicate that the BIV contigs detected in Berlin-Center and Berlin-Zehlendorf were highly conserved across both genomic RNA segments.

Toti-like contigs assigned to the order *Ghabrivirales* and family *Orthototiviridae* according to the current ICTV taxonomy (Simmonds et al., 2024) were detected in both pooled birch pollen datasets. In Berlin-Center, the toti-like signal comprised five birch toti-like virus (BTLV) contigs and two additional divergent toti-like contigs related to Hortaea werneckii totivirus 1 (**Table 1**). The five BTLV contigs showed the highest nt identity to BTLV (acc. no. PP740463.1), with identities ranging from 91.85% to 97.35% at the nt level (data not shown). Specifically, BTLV-12570 (413 nt; coverage 135.14×) showed 97.35% nt identity, BTLV-5268 (853 nt; coverage 580.79×) showed 95.34% nt identity, BTLV-7414 (630 nt; coverage 47.02×) showed 93% nt identity, BTLV-7413 (630 nt; coverage 50.62×) showed 92% nt identity, and BTLV-33227 (239 nt; coverage 1.28×) showed 91.85% nt identity to BTLV-associated sequences. Among the representative BTLV contigs included in **Table 1**, BTLV-33227, corresponding to the RdRP region, showed 91.14% aa identity to the BTLV protein (acc. no. XAI71506.1), whereas BTLV-5268, corresponding to the coat protein region, showed 98.89% aa identity to BTLV coat protein (acc. no. XAI71505.1).

In addition to BTLV, two divergent toti-like contigs were identified exclusively in the Berlin-Center dataset: birch-associated toti-like virus 11152 (452 nt; 2.05× coverage; acc. no. PZ435551) and birch-associated toti-like virus 4326 (993 nt; 2.72× coverage; acc. no. PZ435552). In contrast to the BTLV contigs, both sequences showed relatively low similarity to known totiviruses. Their closest BLASTx matches were to fungal-associated members of the order *Ghabrivirales*, including Hortaea werneckii totivirus 1, whereas the longest contig (4,326 nt) also showed 73.31% nucleotide identity to an unclassified *Ghabrivirales* sequence (acc. no. PV236722) and 65% amino acid identity to the RNA-dependent RNA polymerase of Hortaea werneckii totivirus 1 (acc. no. AZT88647.1) (**Table 1**). Based on their sequence similarity, these contigs are provisionally designated as birch-associated toti-like sequences, a designation that reflects their detection in birch pollen rather than their biological host. Because both sequences are short and partial, neither their taxonomic placement nor their host association can be resolved reliably. Full-length genome sequences and additional biological evidence will be required before their taxonomic status or biological host can be determined.

In Berlin-Zehlendorf, the totivirus-like signals were assigned to a single BTLV associated contig, BTLV-12804 (acc. no. PZ525091; 314 nt), which showed 94.27% nt identity and 97.12% aa identity to BTLV (acc. no. PP740463.1) (**Table 2**).

Ourmia-like sequences assigned to the family *Botourmiaviridae* were detected in both pooled birch pollen datasets. In Berlin-Center, two short contigs were identified (**Table 1**). Birch associated ourmia-like virus 27774 (acc. no. PZ435554; 258 nt), corresponding to an RdRP region, showed 95.74% nt identity to Erysiphe necator associated ourmia-like virus 26 (acc. no. MN611554.1) and 100% aa identity to the corresponding RdRP protein (acc. no. QKI79856.1; 277 nt) (**Table 1**). The second ourmia-like contig from Berlin-Center, birch associated ourmia-like virus 23512, showed 92% nt identity to Plasmopara viticola lesion associated ourmia-like virus 70 (acc. no. NC_076799.1; 277 nt) and 98.90% aa identity to Erysiphe necator associated ourmiavirus-like sequence 26 (**Table 1**). In Berlin-Zehlendorf, one contig, birch associated ourmia-like virus 14815 (acc. no. PZ435553; 233 nt), was detected and showed 95.11% nt identity to Erysiphe necator associated ourmia-like virus 26 and 100% aa identity to Plasmopara viticola lesion associated ourmia-like virus 70 (**Table 2**). Based on their BLASTn and BLASTx identities to members of the family *Botourmiaviridae*, these short RdRP-associated contigs were provisionally designated as birch-associated ourmia-like sequences. This designation reflects their recovery from birch pollen samples and does not imply that birch is the biological host. Given their close similarity to virus sequences reported from fungal- and oomycete-associated datasets, it cannot be ruled out that these contigs originate from pollen, from pollen-colonizing microorganisms or co-extracted microbial material.

Additional virus-like assignments were detected in the Berlin-Center dataset. These included one alphachrysovirus-like contig and one alphaendornavirus-like contig, both represented by single short fragments with low contig coverage (**Tables 1**, **3**). The alphachrysovirus-like contig (GenBank acc. No. PZ525086; 289 nt) showed no significant BLASTn identity but had a BLASTx match to Macrophomina phaseolina chrysovirus 1 (acc. no. YP_009667011.1). The alphaendornavirus-like contig (PZ525089; 233 nt) showed identity to alphaendornavirus-related sequences, including Helianthus annuus alphaendornavirus (acc. no. YP_009553502.1). Because both assignments were supported by only a single short contig, they are conservatively reported as putative virus-like sequences based solely on BLASTn and BLASTx sequence similarity. The top nonredundant BLASTx hits for each provisional birch-associated viral sequence are summarized in **Supplementary Table 5**. An overview of the 45 viral and virus-like sequences generated in this study, including their geographic origins and collection dates, is provided in **Supplementary Table 6**.

### 3.3 *Post-hoc* RT-PCR detection of selected viruses

The presence of CLRV, BIV and BTLV was assessed by virus-specific RT-PCR using RNA extracted from individually processed birch pollen samples collected in 2025 from resampled trees within the original Berlin sampling framework (**Supplementary Figures 1–3**). All of them were detected in the 2025 samples, demonstrating that these viruses remained detectable in birch pollen five years after the original metatranscriptomic survey (**Supplementary Table 7**).

CLRV was detected in one of 11 Berlin-Center samples (9.1%) and seven of eight Berlin-Zehlendorf samples (87.5%) (**Supplementary Table 7**), representing a significant difference in detection frequency between the two sampling areas (Fisher’s exact test, *P* = 0.001). BIV was detected in eight of 11 Berlin-Center samples (72.7%) and seven of eight Berlin-Zehlendorf samples (87.5%), with no significant difference between the two areas (Fisher’s exact test, *P* = 0.603). BTLV was detected in six of 11 Berlin-Center samples (54.5%) and all eight Berlin-Zehlendorf samples (100%). This difference should be interpreted cautiously because of the small sample size and multiple comparisons.

Multiple virus detections were observed in both sampling sites (**Supplementary Table 7**). Simultaneous detection of CLRV, BIV and BTLV occurred in one of 11 Berlin-Center samples (9.1%) and six of eight Berlin-Zehlendorf samples (75.0%) representing a significant difference in co-detection frequency between the two locations (Fisher’s exact test, *P* = 0.006). The Berlin-Center samples showed a more heterogeneous detection pattern, including five samples positive only for BIV, three positive only for BTLV and two positive for both BTLV and BIV. In contrast, the Berlin-Zehlendorf samples were dominated by BTLV-positive pollen, most frequently together with BIV and CLRV.

To provide biological context for the pollen virome detections, the visual leaf status and available leaf RT-PCR results from the corresponding trees were also evaluated. Of the 19 trees resampled in 2025, 15 exhibited virus-like foliar symptoms, including chlorotic spots, mosaic, interveinal chlorosis, leaf rolling, leaf-edge chlorosis, and leaf deformation, whereas four trees appeared asymptomatic. Pollen-associated viruses were detected in both symptomatic and apparently asymptomatic trees. Notably, BTLV, BIV and/or CLRV were detected in pollen from asymptomatic trees, indicating that pollen virus detection in pollen was not restricted to trees exhibiting visible foliar symptoms.

Where corresponding leaf RT-PCR data were available, leaf-associated viruses, including BLRaV, BiCV, and ApMV, had previously been detected in a subset of the corresponding trees (**Supplementary Table 1**). Although several trees tested positive for BLRaV, BiCV and ApMV in leaf tissue, none of these leaf-associated viruses was detected in the corresponding pollen samples, and specifically no BLRaV-associated sequences were recovered from the RNA-based pollen metatranscriptomic datasets. Overall, no consistent relationship was observed between leaf infection status and the pollen virome. For example, one symptomatic tree exhibiting mild interveinal chlorosis and leaf rolling tested negative for all selected leaf viruses, whereas an apparently asymptomatic tree was positive for BLRaV and ApMV in leaves but yielded only BIV in pollen.

### 3.4 Sequence characterization of BTLV

Using specific primers designed from HTS-derived contigs and a primer-walking strategy (**Supplementary Table 2**), a near-complete sequence of the Berlin BTLV pollen-associated sequence E59142 (acc. no. PZ418024) was obtained. After trimming the primer-derived sequences, the final sequence was 4,887 bp in length, representing a near-complete genome compared with the 4,967-bp genome sequence of BTLV isolate E001 from the USA available in GenBank (acc. no. PP740463). Whole-genome comparison showed that the Berlin BTLV pollen-associated sequence E59142 shared 93% nt identity with isolate E001. The deduced coat protein (CP) sequence, which is 2,262 nt long and encodes 754 aa, shared 91% nt identity and 99% aa identity with that of E001. The RdRP, which is 2,499 nt long and encodes 833 aa, shared 93% nt identity and 97% aa identity with the same isolate.

In addition to the HTS-derived contigs, two BTLV consensus sequences were obtained by mapping the pooled pollen reads to the BTLV reference genome. These sequences were designated as birch toti-like virus isolate Berlin-Center (acc. no. PZ435549) and birch toti-like virus isolate Berlin-Zehlendorf (acc. no. PZ435550). Pairwise alignment showed that the Berlin-Center and Berlin-Zehlendorf BTLV consensus sequences shared 99% nt identity. The Berlin-Zehlendorf consensus sequence shared 94% nt identity with both the near full-length BTLV isolate E59142 from this study (acc. no. PZ418024) and the reference BTLV isolate E001 (acc. no. PP740463). The Berlin-Center consensus sequence shared 92% nt identity with both BTLV isolate E59142 and the reference BTLV isolate E001 from the USA. These results indicate that the BTLV sequences recovered from the two pooled pollen datasets were highly similar to each other and closely related to both the Berlin E59142 isolate and the BTLV genome from the USA.

Alignment of the CP coding region revealed stop-codon variation in the termination codon between the Berlin and U.S. BTLV sequences. The three Berlin BTLV sequences (E59142, Berlin-Center, and Berlin-Zehlendorf) terminated with an amber stop codon (TAG), whereas the U.S. isolate E001 terminated with an ochre stop codon (TAA). In contrast, no variation was observed in the RdRP coding region, where all analyzed Berlin and U.S. BTLV sequences terminated with TAG. Similar stop-codon variation has been reported among isolates of other plant RNA viruses including ash shoestring-associated emaravirus (Nouri et al., 2025). Moreover, the identity of the stop codon and its surrounding nucleotide sequence context can influence translation termination or translational readthrough efficiency in viral and eukaryotic systems (Firth and Brierley, 2012; Miras et al., 2017; Ho and Hurst, 2022; Lukhovitskaya et al., 2024). However, whether the CP stop-codon variation observed in BTLV has any functional or biological significance remains unknown and will require experimental investigation.

Conserved motif analysis showed that the deduced RdRP aa sequence of the Berlin BTLV isolate E59142 contained the same eight conserved RdRP motifs characteristic of totiviruses (Khalifa and MacDiarmid, 2019), including the highly conserved catalytic GDD motif, a hallmark of viral RNA-dependent RNA polymerases (Venkataraman et al., 2018). The deduced coat protein also contained the conserved YxDxH motif, represented in BTLV by YSDGH. The presence of this motif in the BTLV CP has been suggested to indicate a possible cap-snatching function (Reyes-Proaño et al., 2024).

### 3.5 Phylogenetic analyses

Phylogenetic analyses were performed separately for CLRV RNA1- and RNA2-associated contigs. Only CLRV contigs longer than 800 nt were included with sufficient overlap within the same genomic region were included to ensure reliable sequence alignment and phylogenetic inference. For RNA1, four contigs (CLRV Berlin-Center 727, 1474, 1685, 1746; GenBank acc. nos. PZ435532, PZ435535, PZ435537 and PZ435538, respectively) met these criteria. The final RNA1 alignment comprised 1,715 nucleotides corresponding to positions 4,692–6,406 of CLRV isolate BpenGer407526 RNA1 (GenBank acc. no. MK402281; 7,848 nt). This region encompasses the downstream portion of RNA1, corresponding predominantly to the C-terminal region of the RdRP coding sequence, together with a short adjacent 3′-terminal region.

**Figure 1:**
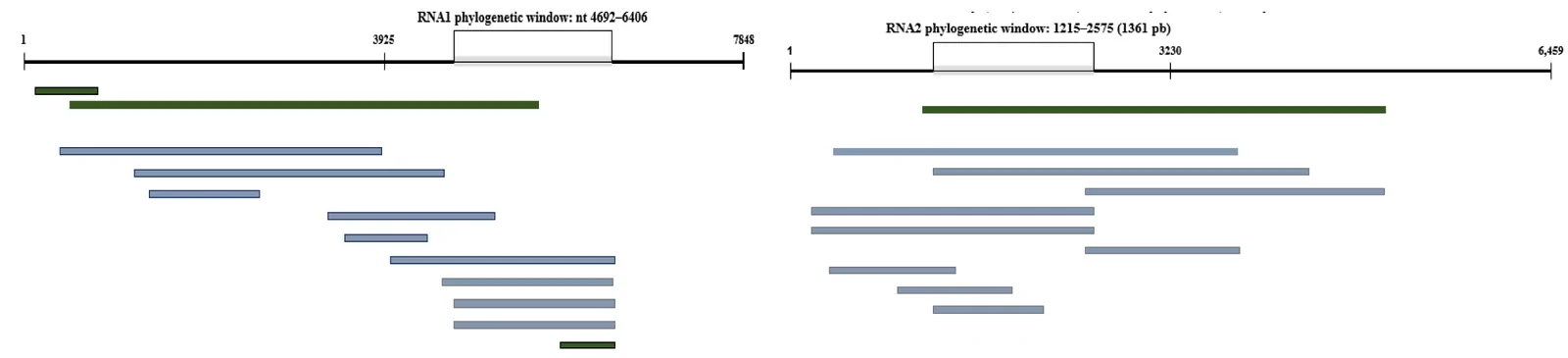
Genome distribution of CLRV-associated contigs recovered from pooled *Betula* sp. pollen samples collected in Berlin. Contigs were mapped according to their BLASTn alignment coordinates against the reference genome of birch CLRV, isolate BpenGer407526 (GenBank accession nos. MK402281 for RNA1 (left) and MK402282 for RNA2 (right)). Contigs derived from the Berlin-Zehlendorf dataset are shown in green, whereas contigs from the Berlin-Center dataset are shown in blue. Contigs shorter than 700 nt are not displayed.

For RNA2, four contigs met the inclusion criteria: CLRV Berlin-Zehlendorf 42 and CLRV Berlin-Center 237, 322, and 765 (GenBank acc. nos. PZ435526, PZ435529, PZ435530 and PZ435533, respectively). The final RNA2 alignment comprised 1,361 nucleotides corresponding to positions 1,215–2,575 of CLRV isolate BpenGer407526 RNA2 (GenBank acc. no. MK402282; 6,459 nt). This region is located within the central part of the RNA2-encoded polyprotein (P2) and predominately encompasses the movement protein region (**Figure 1**).

**Figure 2:**
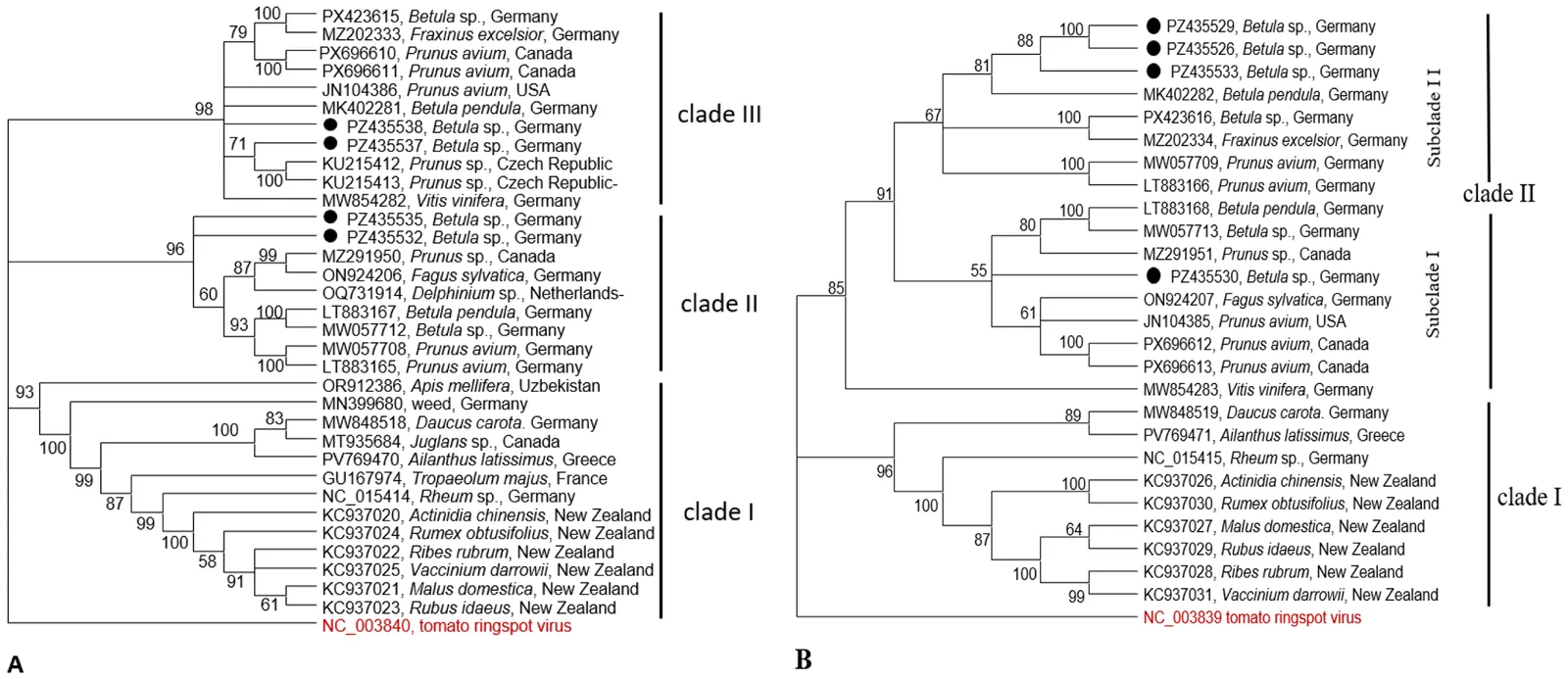
Maximum-likelihood phylogenetic analyses of cherry leaf roll virus (CLRV) based on partial RNA1 and RNA2 sequences. **(A)** Phylogenetic tree inferred from a 1,673-nt region of RNA1. **(B)** Phylogenetic tree inferred from a 1,361-nt region of RNA2. Representative CLRV isolates from diverse host species and geographic origins were included. Multiple sequence alignments were generated using MAFFT v7 with default parameters and maximum-likelihood trees were reconstructed in MEGA 12 using 1,000 bootstrap replicates. The best-fitting nucleotide substitution model, selected according on the Bayesian Information Criterion value, where GTR+G+I for RNA1 and K2+G+I for RNA2. Trees were rooted with the corresponding RNA1 and RNA2 sequences of tomato ringspot virus (ToRSV), shown in red. Bootstrap support values ≥50% are indicated at the nodes. Berlin CLRV sequences identified in the present study are marked with black circles and the major clades and subclades are indicated.

Phylogenetic analysis based on a 1,715-nt region of CLRV RNA1 resolved the analyzed isolates into three well-supported clades, designated Clades I, II, and III (**Figure 2A**). Clade I comprised the most divergent CLRV isolates and included sequences from a wide range of hosts and geographic origins, including *Rheum* sp. from Germany, *Juglans* sp. from Canada, *Ailanthus altissima* from Greece, *Tropaeolum majus* from France, *Daucus carota* from Germany, and several plant-associated isolates from New Zealand, including *Actinidia chinensis*, *Rumex obtusifolius*, *Ribes rubrum*, *Vaccinium darrowii*, *Malus domestica*, and *Rubus idaeus*. This clade corresponds to Group C of the CLRV classification proposed by Rebenstorf et al. (2006). None of the contigs identified in the present study clustered within this clade.

Clade II consisted predominantly of CLRV isolates from woody hosts, including *Prunus* sp. (Canada), *Prunus avium*, *Fagus sylvatica*, *Betula pendula* (Germany), together with a single *Delphinium* sp. (Netherlands). The Berlin-Center contigs 1474 and 727 (GenBank acc. nos. PZ435535 and PZ435532, respectively), were placed within this clade. However, these Berlin-derived contigs remained as independent branches within the clade but formed independent branches rather than a distinct Berlin-specific lineage.

Clade III also comprised predominantly CLRV isolates from woody hosts, including *Betula pendula*, *Fraxinus excelsior*, *Vitis vinifera* from Germany, *Prunus avium* from Canada and the USA, *Prunus* sp. from the Czech Republic. This clade corresponds to Group A of the CLRV classification proposed by Rebenstorf et al. (2006). Within this clade, CLRV Berlin-Center 1685 (GenBank acc. no. PZ435537) formed a distinct branch, whereas CLRV Berlin-Center 1746 (GenBank acc. no. PZ435538) clustered with two *Prunus* sp. isolates from the Czech Republic (GenBank acc. nos. KU215412 and KU215413). As in Clade II, the Berlin-derived contigs did not form a distinct geographic subclade.

The distribution of the Berlin-derived RNA1 contigs across two different CLRV clades demonstrates substantial genetic heterogeneity within the pooled Berlin-Center pollen sample. Rather than forming a single monophyletic Berlin-specific lineage, the contigs were distributed among different phylogenetic groups, indicating the presence of more than one CLRV RNA1 sequence type. Because the analyzed material consisted of pooled pollen collected from multiple trees, this genetic diversity can only be interpreted at the pool level and cannot be attributed to individual trees.

Phylogenetic analysis of a 1,361-nt region of CLRV RNA2 resolved the analyzed isolates into two major clades, designated Clade I and Clade II (**Figure 2B**). Clade I contained the most divergent group of CLRV isolates and corresponded to the divergent Clade I observed in the RNA1-based phylogeny.

Clade I comprised the most divergent CLRV isolates and corresponded to the divergent Clade I identified in the RNA1-based phylogeny, which corresponds to group C based on the classification according to Rebenstorf et al. (2006). None of the RNA2 contigs recovered in the present study clustered within this clade.

Clade II contained the remaining isolates and consisted predominantly of sequences from woody hosts, corresponding to group A according to Rebenstorf et al. (2006). Within this clade, two well-defined subclades were resolved, although the CLRV isolate from *Vitis vinifera* in Germany (GenBank acc. no. MW854283) occupied an independent position outside both subclades. CLRV Berlin-Zehlendorf 42 and CLRV Berlin-Center 237 and 765 (GenBank acc. nos. PZ435526, PZ435529, and PZ435533, respectively) clustered within Subclade II. Among these, CLRV Berlin-Center 237 and Berlin-Zehlendorf 42 were closely related, whereas Berlin-Center 765 formed a separate branch within the same subclade. In contrast, CLRV Berlin-Center 322 (GenBank acc. no. PZ435530) was placed in Subclade I, where it occupied an independent branch. Thus, although all Berlin-derived RNA2 contigs belonged to Clade II, their distribution across two distinct subclades provides further evidence for the presence of multiple CLRV RNA2 sequence types within the pooled Berlin-Center pollen sample.

Comparison of the RNA1 and RNA2 phylogenies revealed that the overall population structure of CLRV was largely conserved between the two genomic regions. In both trees, the most divergent lineage was consistently recovered in Clade I and comprised the same core group of genetically distinct isolates. Likewise, the remaining isolates, predominantly associated with woody hosts, formed related phylogenetic groups in both analyses, although their internal topology differed. Overall, the RNA1 and RNA2 phylogenies supported the same major CLRV lineages, particularly the clear separation of the divergent Clade I from the remaining isolates, while revealing differences in the internal relationships among the woody host-associated lineages.

## 4 Discussion

High-throughput sequencing (HTS) has become a key tool in plant pathology and forest health research because it enables broad, untargeted detection of viral sequences in complex biological samples (Kutnjak et al., 2021; Maina et al., 2024). At the same time, interpretation of HTS data remains challenging, since the detection of viral nucleic acids does not necessarily prove true infection of the sampled host or provide insight into the biological relevance of the discovered virus. This leaves a major gap in linking virus discovery to viral disease epidemiology (Massart et al., 2022; EPPO, 2022). Pollen is not only a plant reproductive structure but also a complex biological microhabitat. Consequently, pollen RNA-Seq datasets may contain plant-derived transcripts alongside RNAs from viruses and from diverse pollen-associated microorganisms, including surface-colonizing and/or potentially endophytic bacteria and fungi (Obersteiner et al., 2016; Manirajan et al., 2018).

In this study, metatranscriptomic sequencing of pooled birch pollen samples from two urban sites in Berlin revealed a diverse set of virus-associated sequences. The most robust detections corresponded to established or recently described birch-associated viruses, including CLRV, BIV, and BTLV, whose continued detectability in Berlin birch pollen was further supported by targeted RT-PCR analysis of individually processed pollen samples. These findings expand the known tissue and geographic distribution of BTLV while confirming the recurrent detection of CLRV and BIV in birch pollen.

The two Berlin sampling areas showed descriptive differences in pollen-associated virus detection patterns. In Berlin-Zehlendorf, the 2025 RT-PCR dataset showed frequent detection of CLRV, BIV, and BTLV, whereas the Berlin-Center samples showed a more heterogeneous pattern with lower CLRV detection. The 2020 pooled HTS datasets also differed descriptively, with Berlin-Center containing a broader set of virus-associated and virus-like contigs.

The CLRV-associated contigs exhibited considerable sequence diversity, with the Berlin-derived sequences distributed across multiple phylogenetic clades or groups, indicating the presence of more than one CLRV variants within the pooled pollen samples. Both the RNA1- and RNA2-based phylogenies revealed substantial genetic heterogeneity, consistent with the population structure previously described for CLRV (Rumbou et al., 2016). In urban birch populations in Rovaniemi, Finland, CLRV populations were likewise found to be highly diverse, with mixed infections involving phylogenetically distinct variants within individual trees and evidence of recombination (Rumbou et al., 2016). However, because the present analyses were based on pooled pollen collected from multiple trees and the RNA1 and RNA2 contigs could not be linked to complete viral genomes or individual hosts, the exact number of CLRV sequence types present could not be determined.

By contrast, the BIV sequences identified in the present study exhibited little sequence divergence and were closely related to previously reported Berlin isolates, indicating that similar BIV variants are associated with birch pollen.

A major finding of this study is the reliable detection and near-complete characterization of BTLV from birch pollen in Germany. BTLV was recently described from *Betula pendula* in the USA, where it was detected in tissue culture-derived and greenhouse-maintained birch plants, suggesting that the virus may have persisted in this material for at least 25 years. Leaf symptoms observed in these plants had previously been attributed to nutrient deficiencies (Reyes-Proaño et al., 2024). The Berlin pollen-derived BTLV isolate E59142 showed high identity to the U.S. leaf-derived BTLV isolate E001 (acc. no. PP740463), with high aa identity in both the CP and RdRP regions. This finding expands the known geographic distribution of BTLV from North America to Europe and extends its detection context from leaf material to pollen material. Together with the previous report from long-maintained birch material, these results suggest that BTLV may have a broad and possibly long-standing association with *Betula*.

The detection of BTLV in birch pollen is particularly noteworthy given its phylogenetic placement within the family *Orthototiviridae*, a group of fungus- and plant-associated toti-like viruses with double-stranded (ds) RNA (Reyes-Proaño et al., 2024). Members of this family are generally associated with persistent infections and several plant-associated dsRNA viruses are considered to be maintained primarily through vertical rather than efficient horizontal transmission (Roossinck, 2018; García-Ordóñez and Pagán, 2024). However, the evolutionary history of these viruses suggests that host associations should be interpreted cautiously. Several dsRNA viruses have been reported from both plants and plant-associated organisms, and incongruence between virus and host phylogenies has been interpreted as evidence of recurrent horizontal or cross-kingdom transmission over evolutionary time (Roossinck, 2018; Andika et al., 2023). Consequently, although BTLV has been consistently detected in both leaf and pollen samples, this does not resolve its biological host. Accordingly, recovery of BTLV from pollen is not evidence of pollen-mediated transmission. Rather, it identifies reproductive tissues as a potentially important compartment in the ecology of BTLV and provides a rationale for future studies. These should include localization of BTLV RNA within reproductive tissues, controlled pollination experiments, and subsequent screening of embryos, seeds, and seedlings to determine whether pollen contributes to virus persistence or vertical transmission.

In addition to these established birch-associated plant viruses, short novel contigs were provisionally assigned to toti-like and ourmia-like lineages. Because these contigs were short and supported by low sequencing depth, they were regarded as tentative virus-like sequences until longer genomic regions and stronger biological evidence become available. Still, the occurrence of these contigs further underscores the complex composition of the pollen virome.

Two additional toti-like sequences were detected that were clearly distinct from BTLV. Their closest relatives were fungus-associated members of the order *Ghabrivirales* (De Coninck et al., 2025), suggesting that these sequences are more likely associated with pollen-colonizing fungi or other co-extracted microorganisms than with birch itself. However, because both sequences were represented by short partial contigs, neither their taxonomic position nor their biological host can be resolved without additional sequence and experimental evidence.

The provisional ourmia-like contigs were evaluated based on sequence identity. These short RdRP-associated fragments showed significant BLASTx identity to members of the family *Botourmiaviridae* previously identified in grapevine virome and grapevine fungal and oomycete pathogens. Specifically, birch-associated ourmia-like sequence 14815 was most closely related to *Botourmiaviridae* sequences reported from *Erysiphe necator* and grapevine-associated samples, whereas sequence 27774 showed highest similarity to virus sequences associated with *Plasmopara viticola* and *Erysiphe necator*. Members of the family *Botourmiaviridae* have been identified in plants, fungi, and oomycetes (Donaire et al., 2024), and viruses of the genus *Ourmiavirus* are characterized by a mosaic evolutionary origin, with different genome segments exhibiting distinct evolutionary affinities (Ayllón et al., 2020).

The proximity of the birch associated ourmia-like viral sequences to viruses previously detected in grapevine, and, at the same time, in the causal agents of grapevine powdery mildew (*E. necator*) and grapevine downy mildew (*P. viticola*), suggests that they belong to viral lineages occurring in complex plant–microorganism systems rather than to clearly host-specific plant viruses (Chiapello et al., 2020). For example, Vitis vinifera RNA virus 1 was identified from grapevine transcriptome data and annotated as a *Botourmiaviridae*-related virus (Chiapello et al., 2020).

Additional alphachrysovirus-like and alphaendornavirus-like fragments, detected only in the Berlin-Center pooled dataset further illustrate the diversity of virus-like sequences recovered from birch pollen. Chrysoviruses, including alphachrysoviruses, are primarily associated with fungi, although chrysovirus-related sequences have also been reported from plant- and insect-associated datasets (Kotta-Loizou et al., 2020). Likewise, alphaendornaviruses have been described from plants, fungi, and oomycetes (Kotta-Loizou et al., 2020). These short fragments may therefore represent RNA originating from the plant itself or from pollen-associated microorganisms. Because each assignment was supported by only a single short contig, they are reported here conservatively as putative virus-associated sequences inferred from sequence identity.

Overall, the present RNA-Seq data indicate that birch pollen harbors a composite virome, including established birch-associated plant viruses as well as - among others - short, low-depth toti-like and ourmia-like sequences whose closest relatives have been reported from fungal- and oomycete-associated systems. Given the presence of microorganisms on pollen surfaces, the most parsimonious explanation is that these provisional contigs may reflect viruses associated with pollen-colonizing fungi, oomycetes, spores, hyphal fragments, or other co-extracted microbial material rather than birch cells themselves. Therefore, these contigs should be interpreted as pollen-associated viral signals, and their biological host remains unresolved.

The pollen-associated virome appears to differ from the virus spectrum previously reported from vegetative or leaf tissues of birch. Studies of birch leaf tissues, including those addressing the birch leaf roll disease (BLRD), have shown that mixed infections are common and often involve plant viruses such as birch leaf roll-associated virus (BLRaV), cherry leaf roll virus (CLRV), apple mosaic virus (ApMV), birch capillovirus (BiCV), and BIV (Opoku et al., 2018; Büttner et al., 2023). Multiple viral infections in individual birch trees have also been reported (Opoku et al., 2018; Opoku, 2023; Rumbou et al., 2020), suggesting that BLRD-related symptom expression may reflect the presence of a complex virome rather than the effect of a single virus alone (Rumbou et al., 2020). Consequently, attributing specific symptoms to individual viruses remains difficult without additional experimental evidence.

The birch pollen virome characterized in this study consisted exclusively of RNA viruses and RNA virus-like sequences. This finding is consistent with previous studies showing that pollen-associated viromes are dominated by RNA viruses, whereas DNA viruses are reported much less frequently (Fetters et al., 2022; Fetters and Ashman, 2023). This pattern likely reflects both biological and methodological factors. From a biological perspective, most currently recognized pollen-associated and pollen-transmitted plant viruses possess RNA genomes, while pollen transmission appears to be less common among plant DNA viruses. Methodologically, most pollen virome studies rely on RNA sequencing or metatranscriptomic approaches, which are highly effective for detecting RNA viruses but identify DNA viruses only indirectly through their expressed transcripts. Consequently, DNA viruses that are transcriptionally inactive or expressed at low levels in pollen may remain undetected.

Within the vegetative birch virome, birch leaf roll-associated virus (BLRaV), a DNA pararetrovirus belonging to the genus *Badnavirus* (family *Caulimoviridae*), is considered as the main causal agent of birch leaf roll disease (BLRD), which is widely distributed across Europe (von Bargen et al., 2009; Rumbou et al., 2018; 2020). BLRaV-associated sequences were detected in the birch pollen metatranscriptomes analyzed in the present study. However, the absence of BLRaV-derived RNA should not be interpreted as definitive evidence that the virus is absent from pollen. Because the study employed an RNA-based approach, transcriptionally inactive DNA viruses present in pollen would not be detected. Thus, the lack of a BLRaV signal may reflect the absence of viral transcription rather than the absence of viral DNA.

Determining whether BLRaV is genuinely absent from birch pollen or simply undetectable by RNA sequencing therefore warrants further investigation. Although pollen transmission is not considered the predominant transmission route for most badnaviruses, several members of the genus have been reported to be seed- or pollen-associated (Bhat et al., 2023). For example, cacao swollen shoot virus (CSSV) DNA has been detected in pollen collected from infected cacao trees, although controlled pollination experiments provided no evidence for transmission to seeds or seedlings (Ameyaw et al., 2013). Likewise, taro bacilliform virus (TaBV) has been detected in pollen from taro flowers (Macanawai et al., 2005). These observations demonstrate that badnavirus DNA can occur in pollen but do not establish pollen-mediated transmission. To distinguish true absence of BLRaV from the absence of detectable viral transcripts, targeted DNA-based analyses of birch pollen should be performed. Even if BLRaV DNA were detected, its biological significance would require careful interpretation, as it could represent episomal viral DNA, surface contamination, or endogenous badnavirus-like sequences rather than active infection of pollen cells or evidence of pollen-mediated transmission (Bhat et al., 2023).

The detection of plant viruses in pollen should not be interpreted automatically as evidence of active viral replication within pollen tissue. Pollen-associated viruses may occur externally on the pollen surface or internally within pollen grains and pollen tubes, where they may contribute to horizontal or vertical transmission (Fetters and Ashman, 2023). Accordingly, wild-plant pollen viromes have been described as potential vehicles for viral dissemination, with viruses being transported on or within pollen grains by pollinators (Fetters et al., 2022). Importantly, the term *pollen-associated* encompasses a broader range of biological scenarios than *actively replicating in pollen*. Consequently, the pollen-associated virome should be regarded as a distinct component of the birch-associated virome rather than as a direct proxy for the viromes of leaves, bark, or other vegetative tissues. Future studies integrating matched pollen, leaf, and pollen-associated microbial samples from the same trees, together with complementary DNA- and RNA-based approaches, will be required to determine whether individual virus-associated sequences represent systemic birch infections, pollen-mediated dispersal, or viruses associated with pollen-colonizing microorganisms.

The combined use of metatranscriptomics and individual-tree RT-PCR performed in the present study highlights both the strengths and limitations of untargeted pollen virome analysis. Because the two datasets differed in both sampling design and sampling year, the RT-PCR results should not be regarded as direct technical validation or quantitative confirmation of the pooled HTS data. The metatranscriptomic libraries were generated from pooled pollen samples collected in 2020, whereas RT-PCR assays were performed on individually processed pollen samples collected in 2025 from resampled trees where possible. Consequently, differences between HTS-derived virus signals and RT-PCR detection frequencies may reflect pooling effects, temporal variation, differences in sequencing depth, and within-tree sampling heterogeneity. Accordingly, contig number and sequence coverage in pooled metatranscriptomic datasets are best interpreted as indicators of virus detection rather than measures of virus prevalence or abundance. In contrast, the RT-PCR analyses provide independent longitudinal evidence that CLRV, BIV, and BTLV remained detectable in birch pollen within the Berlin sampling framework five years after the original HTS survey.

The detection of CLRV, BIV, and BTLV in birch pollen is relevant in the broader context of viral dispersal through reproductive tissues. Pollen- and seed-associated transmission has long been recognized for CLRV in birch (Massalski and Cooper, 1984; Massalski et al., 1988; Card et al., 2007) and the detection of CLRV sequences in both pooled pollen datasets is consistent with this established epidemiological role. Pollen association has also recently been addressed for BIV (Gilles et al., 2023), although its natural transmission route remains unresolved. By contrast, the detection of BTLV in birch pollen provides the first evidence that this recently described toti-like virus can also be associated with the reproductive compartment of birch. However, the presence of BTLV RNA in pollen-derived material alone does not demonstrate pollen-mediated transmission, infection of pollen grains or transmission to progeny.

This cautious interpretation is further supported by the evolutionary context of plant- and fungi-associated persistent viruses. Related dsRNA virus lineages have been reported from both plants and plant-associated fungi, and incongruence between virus and host phylogenies has been interpreted as evidence of recurrent horizontal or cross-kingdom transmission over evolutionary time (Roossinck, 2018; Andika et al., 2023). Therefore, although the phylogenetic placement of BTLV among plant-associated toti-like viruses supports a possible association with birch, it does not resolve the actual biological host, host range, or transmission route. Accordingly, recovery of BTLV from pollen is not evidence of pollen-mediated transmission by itself, but it is a biologically meaningful observation that motivates future studies on BTLV ecology, persistence, tissue localization, and possible vertical transmission.

Overall, these findings broaden our understanding of the virome associated with reproductive tissues of woody trees and emphasize that pollen represents a distinct ecological compartment rather than simply reflecting the virome of vegetative tissues. As forest and urban tree viromes remain far less explored than those of agricultural crops, characterization of pollen-associated viromes provides new opportunities to investigate virus ecology, tissue tropism, and potential transmission pathways in forest ecosystems. Moreover, the detection of both plant-associated viruses and virus-like sequences related to fungal and oomycete viruses supports the tree holobiont concept, in which the host plant and its associated microbiota function as an integrated biological system (Faure et al., 2018; Simon et al., 2019). From this perspective, plant viruses, mycoviruses, and other microbiome-associated viruses should be considered components of a broader tree-associated virome rather than isolated entities (Rumbou et al., 2021; Vainio et al., 2024). Comparative studies across additional tree species and tissues, integrating metatranscriptomics with targeted molecular and experimental approaches, will be essential to elucidate the diversity, ecological roles, and transmission biology of viruses within forest ecosystems.

## Data Availability

The sequence data have been submitted to GenBank with the accession numbers PZ435517–PZ435554, PZ525086–PZ525091, and PZ418024.
