# Supplementary material for: Virome metatranscriptomic profiling of birch pollen reveals a diverse viral community
Source: Front Plant Sci. 2026 Aug 31;17:1897449. doi: 10.3389/fpls.2026.1897449 (PMC13572724; doi:10.3389/fpls.2026.1897449)
Supplement: Supplementary file 1 [file SupplementaryFile1.docx]

M	1	2	3	4	nc

500bp

**Supplementary Figure 1**. An exemplary 1% agarose gel electrophoresis detection of birch toti-like virus (BTLV). RT-PCR products: line 1–4 = E59137, E59142, E59149, E59135. nc = negative control (E59137). Arrows indicate the size of individual bands of the standard. M: 1Kb Gene Ruler DNA Ladder (Thermo Scientific™ SM0311).
