# Supplementary material for: Virome metatranscriptomic profiling of birch pollen reveals a diverse viral community
Source: Front Plant Sci. 2026 Aug 31;17:1897449. doi: 10.3389/fpls.2026.1897449 (PMC13572724; doi:10.3389/fpls.2026.1897449)
Supplement: Supplementary file 2 [file SupplementaryFile2.docx]

M	1	2	3	4	5	6	7	8	nc

500bp

**Supplementary Figure 2**. An exemplary 1% agarose gel electrophoresis detection of cherry leaf roll virus (CLRV). RT-PCR products: line 1–8 = E59142, E59144, E59149, E59150, E59152, E59154, E59159, E59169. nc = negative control (E59120). Arrows indicate the size of individual bands of the standard. M: 1Kb Gene Ruler DNA Ladder (Thermo Scientific™ SM0311).
