# Supplementary material for: Virome metatranscriptomic profiling of birch pollen reveals a diverse viral community
Source: Front Plant Sci. 2026 Aug 31;17:1897449. doi: 10.3389/fpls.2026.1897449 (PMC13572724; doi:10.3389/fpls.2026.1897449)
Supplement: Supplementary file 4 [file Table1.docx]

**Supplementary Table 1**: Pollen samples collected from individual birch trees in Berlin, Germany, including their allocation to pooled HTS datasets and individual RT-PCR analyses, the observed leaf symptom categories, and the infection status of the corresponding trees for BLRaV, BiCV, and ApMV.

| Birch Tree ID | Sampling location (Berlin district) | Host plant | Sample material | Included in pooled HTS analysis (2020) | HTS pool ID (2020) | Sample format for RT-PCR detection (2025) | Leaf symptoms (2020) | Leaf infection status (2020) |
| --- | --- | --- | --- | --- | --- | --- | --- | --- |
| E59119 | Kreuzberg | *Betula* sp. | Pollen | Yes | Berlin-Center | Individual | No symptom | None |
| E59120 | Köpenick | *Betula* sp. | Pollen | Yes | Berlin- Center | Individual | Chlorotic spots | BiCV only |
| E59121 | Köpenick | *Betula* sp. | Pollen | Yes | Berlin- Center | Individual | Chlorotic spots, mosaic | BiCV only |
| E59123 | Tempelhof | *Betula* sp. | Pollen | Yes | Berlin- Center | Individual | Chlorotic spots | BLRaV + ApMV |
| E59124 | Kreuzberg | *Betula* sp. | Pollen | Yes | Berlin- Center | Individual | Faint interveinal chlorosis, leaf roll | None |
| E59125 | Kreuzberg | *Betula* sp. | Pollen | Yes | Berlin- Center | Individual | No symptom | None |
| E59127 | Mitte | *Betula* sp. | Pollen | Yes | Berlin- Center | Individual | Chlorotic spots | BLRaV |
| E59128 | Mitte | *Betula* sp. | Pollen | Yes | Berlin- Center | NI | NT | NT |
| E59129 | Mitte | *Betula* sp. | Pollen | Yes | Berlin- Center | NI | NT | NT |
| E59130 | Pankow | *Betula* sp. | Pollen | Yes | Berlin- Center | NI | NT | NT |
| E59131 | Pankow | *Betula* sp. | Pollen | Yes | Berlin- Center | Individual | Chlorotic spots | BLRaV + ApMV |
| E59135 | Friedrichshain | *Betula* sp. | Pollen | Yes | Berlin- Center | Individual | Leaf mosaic, interveinal chlorosis | BLRaV + ApMV |
| E59137 | Neukölln | *Betula* sp. | Pollen | Yes | Berlin- Center | Individual | Interveinal chlorosis, leaf deformation, chlorotic spots | BLRaV + ApMV |
| E59138 | Neukölln | *Betula* sp. | Pollen | Yes | Berlin- Center | Individual | No symptom | BLRaV + ApMV |
| E59142 | Zehlendorf | *Betula* sp. | Pollen | Yes | Berlin-Zehlendorf | Individual | Slight interveinal chlorosis | NT |
| E59144 | Zehlendorf | *Betula* sp. | Pollen | Yes | Berlin-Zehlendorf | Individual | No symptom | NT |
| E59145 | Zehlendorf | *Betula* sp. | Pollen | Yes | Berlin-Zehlendorf | NI | NT | NT |
| E59146 | Zehlendorf | *Betula* sp. | Pollen | Yes | Berlin-Zehlendorf | NI | NT | NT |
| E59149 | Zehlendorf | *Betula* sp. | Pollen | Yes | Berlin-Zehlendorf | Individual | leaf roll | NT |
| E59150 | Zehlendorf | *Betula* sp. | Pollen | Yes | Berlin-Zehlendorf | Individual | Mosaic, interveinal chlorosis, leaf edge chlorosis | NT |
| E59151 | Zehlendorf | *Betula* sp. | Pollen | Yes | Berlin-Zehlendorf | NI | NT | NT |
| E59152 | Zehlendorf | *Betula* sp. | Pollen | Yes | Berlin-Zehlendorf | Individual | Slight leaf roll | NT |
| E59154 | Zehlendorf | *Betula* sp. | Pollen | Yes | Berlin-Zehlendorf | Individual | Slight leaf roll | NT |
| E59159 | Zehlendorf | *Betula* sp. | Pollen | Yes | Berlin-Zehlendorf | Individual | Interveinal chlorosis | NT |
| E59169 | Zehlendorf | *Betula* sp. | Pollen | Yes | Berlin-Zehlendorf | Individual | No symptom | NT |

**Abbreviations:** HTS, high-throughput sequencing; RT-PCR, reverse transcription–polymerase chain reaction; BiCV, birch carlavirus; ApMV, apple mosaic virus; BLRaV, birch leaf roll-associated virus; NT, not tested; None, no virus detected; NI, not included.

**Note:** NI indicates samples that were included in the pooled HTS analysis in 2020 but were unavailable for individual RT-PCR confirmation in 2025. The tree corresponding to sample E59151 had been cut down. Samples E59128, E59129, E59130, E59145, and E59146 could not be recollected because suitable flowering male catkins were unavailable and the tree crowns could not be accessed safely.
