# Supplementary material for: Virome metatranscriptomic profiling of birch pollen reveals a diverse viral community
Source: Front Plant Sci. 2026 Aug 31;17:1897449. doi: 10.3389/fpls.2026.1897449 (PMC13572724; doi:10.3389/fpls.2026.1897449)
Supplement: Supplementary file 5 [file Table2.docx]

**Supplementary Table 2.** Primers used for near full-length genome amplification of BTLV and for RT-PCR detection of BTLV, BIV, CLRV, BLRaV, ApMV, and BiCV.

| Primer name | Primer sequence (5′ → 3′) | Amplicon size (bp) | Target virus | Purpose | Target region | Reference |
| --- | --- | --- | --- | --- | --- | --- |
| BTLV-55-f | TCGTGAAAAGATTTTTTCCGGAGT | 1166 | BTLV | Genome amplification | Coat protein | This study |
| BTLV-1221-r | ATCTAACCGCCATGCCTG |  |  |  |  |  |
| BTLV-1204-f | CAGGCATGGCGGTTAGATCTG | 558 | BTLV | Genome amplification,  RT-PCR confirmation | Coat protein | This study |
| BTLV-1762-r | GGGAACGGACTCAAAGGCATC |  |  |  |  |  |
| BTLV-1464-f | GAGAAGATAAAGGAAGAGATGACG | 1608 | BTLV | Genome amplification | CP–RdRP overlapping fragment | This study |
| BTLV-3072-r | GCGACAATATATGCCACTTGTG |  |  |  |  |  |
| BTLV-2792-f | AGAAGAGAATTTTCTGTGGACAG | 1409 | BTLV | Genome amplification | RdRp | This study |
| BTLV-4261-r | TGAATGTACCATTGTTGCTGC |  |  |  |  |  |
| BTLV-4098-f | CTAAGATACATAAGCAGATGGCTG | 869 | BTLV | Genome amplification | RdRP | This study |
| BTLV-4967-r | GCTAAGTAGTAATCCGCCAC |  |  |  |  |  |
| Idaeo00001FW | GGGTCATCTTCGGGGAAGTG | 556 | BIV | RT-PCR confirmation | RNA1-replicase | Gilles et al., 2023 |
| Idaeo00001RW | AAAGTCTCCAGCATGACGC |  |  |  |  |  |
| CLRV-RW1 FW | GTCGGAAAGATTACGTAAAAGG | 353 | CLRV | RT-PCR confirmation | 3′ UTR | Gilles et al., 2023 |
| CLRV-RW2mod RV | CATGCGACCGGTCCTAGTAGTA |  |  |  |  |  |
| BadnaSG Forward | ACGAAGAAGGATACCTCACGG | 242 | BLRaV | RT-PCR detection | Coat protein | unpublished/in-house primers |
| BadnaSG Reverse | CCAGTGTCTAGTACCGCCC |  |  |  |  |  |
| BadnaK Forward | GCAGGCACAGAACAATGG | 300 | BLRaV | RT-PCR detection | Coat protein | unpublished/in-house primers |
| BadnaK Reverse | GGCATTACTAGCCATTCGTA |  |  |  |  |  |
| Carla F | GGCACTTACGTACAGGAGT | 197 | BiCV | RT-PCR detection | Coat protein | unpublished/in-house primers |
| Carla R | GCGGAAAAGGGGCTTAGATA |  |  |  |  |  |
| CLRV-CP350F | CATRGAGAAGCTAAATTTCTCTCT | 627 | CLRV | RT-PCR detection | Coat protein | unpublished/in-house primers |
| CLRV-CP977R | ACTCMACCCTATCAAARTATAYCA |  |  |  |  |  |
| CLRV-CP188F | TGTCTGTRAATATTATGGCTGGTAC | 170 | CLRV | RT-PCR detection | RNA1-Polyprotein | von Bargen et al., 2009 |
| CLRV-CP350R | AGAGAGAAATTTAGCTTCTCYATG |  |  |  |  |  |
| ApMV-CP1535F | GTAATCCGAAAGGTCCGAAT | 204 | ApMV | RT-PCR detection | Coat protein | unpublished/in-house primers |
| ApMV-CP1739R | GGTGGTAACTCACTCGTTAT |  |  |  |  |  |

**Abbreviations**: BTLV, birch toti-like virus; BIV, birch idaeovirus; CLRV, cherry leaf roll virus; BiCV, birch carlavirus; ApMV, apple mosaic virus; BLRaV, birch leaf roll-associated virus; RdRP, RNA-dependent RNA polymerase; CP, coat protein; 3′ UTR, 3′ untranslated region.
