# Supplementary material for: Virome metatranscriptomic profiling of birch pollen reveals a diverse viral community
Source: Front Plant Sci. 2026 Aug 31;17:1897449. doi: 10.3389/fpls.2026.1897449 (PMC13572724; doi:10.3389/fpls.2026.1897449)
Supplement: Supplementary file 6 [file Table3.docx]

**Supplementary Table 3**. Summary of high-throughput sequencing results and *de novo* assembly statistics for pooled birch pollen samples collected at two urban sites in Berlin, Germany

.

| Metric | Berlin-Center | Berlin-Zehlendorf |
| --- | --- | --- |
| Raw reads | 17,170,824 | 13,679,796 |
| Filtered paired-end reads | 17,147,307 | 13,658,939 |
| Number of contigs | 108,362 | 79,741 |
| Contig length range (bp) | 100–11,457 | 100–16,500 |
| Mean contig length (bp) | 266 | 249 |
| N50 (bp) | 288 | 264 |
| Total assembly length (bp) | 28,861,143 | 19,862,594 |
