# Supplementary material for: Virome metatranscriptomic profiling of birch pollen reveals a diverse viral community
Source: Front Plant Sci. 2026 Aug 31;17:1897449. doi: 10.3389/fpls.2026.1897449 (PMC13572724; doi:10.3389/fpls.2026.1897449)
Supplement: Supplementary file 7 [file Table4.docx]

**Supplementary Table 4**. Reference-based mapping statistics for selected virus- and virus-like sequence signals identified during the initial CLC Viral RefSeq screening of the Berlin-center and Berlin-Zehlendorf birch pollen datasets.

| Virus or  virus-like signal | Dataset | Reference sequence for CLC screening | RefSeq accession | Reference length (nt) | Consensus length (nt) | Reference covered (%) | Total reads | Mapped reads | Properly paired reads | Mean read depth | Map GenBank acc. No. | nt identity % | Aligned length | Query coverage % |
| --- | --- | --- | --- | --- | --- | --- | --- | --- | --- | --- | --- | --- | --- | --- |
| CLRV RNA1 | Berlin-Zehlendorf | cherry leaf roll virus RNA1 | NC_015414 | 7,918 | 5,080 | 64.16 | 6,280 | 2,644 | 1,652 | 45.57 | LT883167 | 92 | 1,910 | 77 |
| CLRV RNA2 | Berlin-Zehlendorf | cherry leaf roll virus RNA2 | NC_015415 | 6,360 | 3,222 | 50.66 | 3,685 | 1,064 | 638 | 22.34 | MK402282 | 93 | 4,571 | 85 |
| CLRV RNA1 | Berlin-center | cherry leaf roll virus RNA1 | NC_015414 | 7,918 | 6,309 | 79.68 | 28,262 | 12,516 | 7,906 | 217.19 | MZ202333 | 94 | 5,951 | 99 |
| CLRV RNA2 | Berlin-center | cherry leaf roll virus RNA2 | NC_015415 | 6,360 | 4,387 | 68.98 | 15,487 | 5,924 | 3,694 | 126.74 | MZ202334 | 94.49 | 4,754 | 90 |
| BIV-related signal | Berlin-Zehlendorf | privet leaf blotch-associated virus RNA1 | NC_031341 | 5,377 | 645 | 12.00 | 13,789 | 205 | 122 | 4.11 | MK402235 | 99.51 | 5,074 | 96 |
| BIV-related signal | Berlin-center | privet leaf blotch-associated virus RNA1 | NC_031341 | 5,377 | 568 | 10.56 | 17,515 | 162 | 88 | 2.98 | MK402235 | 99 | 5,074 | 96 |
| BTLV/totivirus-related signal | Berlin-Zehlendorf | panax notoginseng virus B | NC_040551 | 4,993 | 298 | 5.97 | 1,085 | 23 | 6 | 0.30 | PP740463 | 95 | 4,929 | 100 |
| BTLV/totivirus-related signal | Berlin-center | panax notoginseng virus A | NC_029096 | 5,003 | 669 | 13.37 | 89,348 | 1,626 | 538 | 0.30 | PP740463 | 95 | 4,918 | 99 |

CLRV: cherry leaf roll virus; BIV: birch idaeovirus; BTLV: birch toti-like virus. E-values for all pairwise comparisons were 0.0.
