# Supplementary material for: Virome metatranscriptomic profiling of birch pollen reveals a diverse viral community
Source: Front Plant Sci. 2026 Aug 31;17:1897449. doi: 10.3389/fpls.2026.1897449 (PMC13572724; doi:10.3389/fpls.2026.1897449)
Supplement: Supplementary file 8 [file Table5.docx]

**Supplementary Table 5**. Summary of the top three nonredundant BLASTx hits for each provisional birch-associated virus-like sequence.

| **No** | **Provisional virus name** | **GenBank accession number** | **BLAST hit accession number** | **Virus/top hit** | **Family** | **Protein** | **aa identity %** | **Aligned length (aa)** | **Query coverage %** | **E-value** | **Hit score** |
| --- | --- | --- | --- | --- | --- | --- | --- | --- | --- | --- | --- |
| **1** | birch-associated toti-like virus | PZ435551 | AZT88647.1 | Hortaea werneckii totivirus 1 | *Orthototiviridae* | RdRP | 67.33 | 150 | 99 | 1e-66 | 227 |
|  |  |  | ADO60925.1 | Grapevine associated mycovirus-1 |  | RdRP | 62.25 | 151 | 99 | 4e-61 | 204 |
|  |  |  | YP_009342434.1 | Wuhan insect virus 27 |  | hypothetical protein | 60.67 | 150 | 99 | 2e-60 | 210 |
| **2** | birch-associated toti-like virus | PZ435552 | AZT88647.1 | Hortaea werneckii totivirus 1 | *Orthototiviridae* | RdRP | 63.16 | 304 | 100 | 2e-129 | 397 |
|  |  |  | UVG55926.1 | Poaceae Liege totivirus 3 |  | RdRP | 50.66 | 304 | 100 | 5e-98 | 317 |
|  |  |  | UYL95666.1 | Nanning Totiv tick virus 3 |  | RdRP | 52.63 | 304 | 100 | 1e-94 | 310 |
| **3** | birch-associated ourmia-like virus | PZ435553 | QKI79870.1 | Erysiphe necator associated ourmia-like virus 40 | *Botourmiaviridae* | RdRP | 100.00 | 95 | 99 | 7e-57 | 196 |
|  |  |  | WVS33812.1 | Vitis vinifera RNA virus 1 |  | RdRP | 75.27 | 93 | 97 | 2e-39 | 147 |
|  |  |  | WPR16980.1 | Sjack associated botourmia-like virus 14 |  | RdRP | 62.77 | 94 | 98 | 2e-36 | 130 |
| **4** | birch-associated ourmia-like virus | PZ435554 | QKI79856.1 | Erysiphe necator associated ourmia-like virus 26 | *Botourmiaviridae* | RdRP | 100.00 | 85 | 99 | 6e-50 | 140 |
|  |  |  | YP_010800264.1 | Plasmopara viticola lesion associated ourmia-like virus 70 |  | RdRP | 98.82 | 85 | 99 | 8e-50 | 176 |
| **5** | birch-associated ourmia-like virus | PZ525090 | YP_010800264.1 | Plasmopara viticola lesion associated ourmia-like virus 70 | *Botourmiaviridae* | RdRP | 98.90 | 91 | 99 | 2e-54 | 189 |
|  |  |  | QKI79853.1 | Erysiphe necator associated ourmia-like virus 23 |  | RdRP | 83.52 | 91 | 99 | 4e-45 | 164 |
|  |  |  | WPR16728.1 | Dracophyllum associated botourmia-like virus 71 |  | RdRP | 60.92 | 87 | 94 | 2e-28 | 114 |
| **6** | birch-associated alphachrysovirus-like | PZ525086 | YP_009667011.1 | Macrophomina phaseolina chrysovirus 1 | *Chrysoviridae* | P4 Movement protein | 35.79 | 95 | 99 | 1e-19 | 72.0 |
|  |  |  | WPA89355.1 | Aspergillus ochraceopetaliformis chrysovirus 1 |  | hypothetical protein | 37.21 | 86 | 89 | 6e-11 | 68.9 |
|  |  |  | YP_009507947.1 | Verticillium dahliae chrysovirus 1 |  | protease | 39.73 | 73 | 76 | 1e-10 | 67.8 |
| **7** | birch-associated alphaendornavirus-like | PZ525089 | QYF50052.1 | Sichuan alphaendornavirus 2 | *Endornaviridae* | polyprotein | 79.03 | 62 | 80 | 3e-25 | 107 |
|  |  |  | WKK30317.1 | Helianthus annuus alphaendornavirus |  | polyprotein | 65.75% | 73 | 94 | 3e-24 | 105 |
|  |  |  | QGW52049.1 | Alphaendornavirus sp. |  | polyprotein | 56.58% | 76 | 98 | 7e-21 | 96.3 |
