# Supplementary material for: Virome metatranscriptomic profiling of birch pollen reveals a diverse viral community
Source: Front Plant Sci. 2026 Aug 31;17:1897449. doi: 10.3389/fpls.2026.1897449 (PMC13572724; doi:10.3389/fpls.2026.1897449)
Supplement: Supplementary file 9 [file Table7.docx]

**Supplementary Table 7.** RT-PCR detection of CLRV, BIV, and BTLV in individual birch pollen samples collected in 2025, together with the corresponding leaf symptom status and status of virus detection in leaf samples (for BLRaV, ApMV and BiCV) of the sampled trees.

| Sample ID | Site | CLRV (pollen) | CLRV (leaf) | BIV (pollen | BIV (leaf) | BTLV (pollen) | BTLV (leaf) | BLRaV (pollen) | BLRaV (leaf) | ApMV (pollen) | ApMV (leaf) | BiCV(pollen) | BiCV (leaf) | Leaf symptoms at pollen sampling timepoint |
| --- | --- | --- | --- | --- | --- | --- | --- | --- | --- | --- | --- | --- | --- | --- |
| E59119 | Center | − |  | − |  | + |  |  |  |  |  |  |  | No symptom |
| E59120 | Center | − |  | − |  | + |  |  |  |  |  |  | + | Chlorosis |
| E59121 | Center | − |  | + |  | + |  |  |  |  |  |  | + | Vein chlorosis, interveinal chlorisis |
| E59123 | Center | − |  | + |  | − |  |  | + |  | + |  |  | Oak leaf pattern, vein banding |
| E59124 | Center | − |  | + |  | − |  |  |  |  |  |  |  | interveinal chlorosis, leaf roll |
| E59125 | Center | − |  | + |  | − |  |  |  |  |  |  |  | No symptom |
| E59127 | Center | − |  | + |  | + |  |  | + |  |  |  |  | Chlorosis |
| E59131 | Center | − |  | − |  | + |  |  | + |  | + |  |  | Oak leaf pattern |
| E59135 | Center | + |  | + |  | + |  |  | + |  | + |  |  | Interveinal/intercostal chlorosis, leaf roll |
| E59137 | Center | − |  | + |  | − |  |  | + |  | + |  |  | Interveinal chlorosis, leaf deformation |
| E59138 | Center | − |  | + |  | − |  |  | + |  | + |  |  | No symptom |
| E59142 | Zehlendorf | − |  | + |  | + |  |  |  |  |  |  |  | interveinal chlorosis |
| E59144 | Zehlendorf | + |  | + |  | + |  |  |  |  |  |  |  | No symptom |
| E59149 | Zehlendorf | + |  | − |  | + |  |  |  |  |  |  |  | interveinal chlorosis, leaf roll |
| E59150 | Zehlendorf | + |  | + |  | + |  |  |  |  |  |  |  | Interveinal/intercostal chlorosis |
| E59152 | Zehlendorf | + |  | + |  | + |  |  |  |  |  |  |  | light interveinal chlorosis, leaf roll |
| E59154 | Zehlendorf | + |  | + |  | + |  |  |  |  |  |  |  | light interveinal chlorosis, leaf roll |
| E59159 | Zehlendorf | + |  | + |  | + |  |  |  |  |  |  |  | Interveinal chlorosis |
| E59169 | Zehlendorf | + |  | + |  | + |  |  |  |  |  |  |  | No symptom |

+: detected by virus-specific RT-PCR; −: not detected by virus-specific RT-PCR. CLRV: cherry leaf roll virus; BIV: birch idaeovirus; BTLV: birch toti-like virus;

BLRaV: birch leaf roll-associated virus; ApMV: apple mosaic virus; BiCV: birch carlavirus; empty cells: not tested.
